# The impact of COVID-19 on the mental health of pregnant women in Shanghai, China

**DOI:** 10.3389/fpubh.2022.938156

**Published:** 2022-10-06

**Authors:** Jiali Zhang, Hualong Yuan, Liping Xu, Chuntao Yi, Weiming Tang

**Affiliations:** ^1^Fenglin Community Health Service Center, Shanghai, China; ^2^Guangdong Second Provincial General Hospital, Guangzhou, China; ^3^The University of North Carolina at Chapel Hill Project-China, Guangzhou, China

**Keywords:** depression, anxiety, pregnant women, COVID-19, China

## Abstract

**Background:**

COVID-19 has dramatically impacted people's health, especially mental health. This study aimed to compare the psychological status of pregnant women before and after the COVID-19 outbreak.

**Methods:**

Participants were recruited (from September 29, 2019, to November 5, 2020) and screened by the Patient Health Questionnaire (PHQ-9) and the Generalized Anxiety Disorder Scale (GAD-7). The study participants were categorized into three groups based on two turning-points: January 23, 2020, when China initiated a locked-down strategy, and May 11, 2020, when Shanghai started to ease the COVID-19 measures. Multivariable logistic regression was used to determine the factors associated with depression and anxiety in pregnant women. We used enter method for variable selection; only variables with *P* <0.10 were included in the final model.

**Results:**

We recruited 478 pregnant women. After the outbreak, the depression rate (PHQ-9 ≥ 5) increased by 12.3% (from 35.4 to 47.7%), and the anxiety rate (GAD-7 ≥ 5) was stable (13.3 vs. 16.2%). The multivariable logistic regression results further confirmed that the odds of depression in pregnant women increased 81% after the outbreak (aOR = 1.81, 95%CI: 1.16–2.84). However, the median depression scale score was still statistically higher after the pandemic situation was stable (5.0 vs. 4.0) compared to the outbreak period.

**Conclusion:**

The depression rate increased among pregnant women after the outbreak and was not recovered after the ease of COVID-19 measures in Shanghai. Health institutes should pay attention to the long-term influence of the pandemic.

## Introduction

By May 6, 2022, 2,284,491 confirmed cases of COVID-19, and 14,497 deaths had been reported in China (1). Globally, as of May 6, 2022, there have been 516,607,318 confirmed cases of COVID-19, including 6,249,223 deaths (2). COVID-19 is the second new infectious disease declared the “global pandemic” by the World Health Organization (3) and was considered the biggest pandemic in the twenty-first century. The pandemic has strongly shifted the world and greatly impacted people's health, significantly increasing the burden of mental health illnesses (4).

Pregnancy is a particular period associated with physiological changes, making pregnant women more susceptible to respiratory infections, such as COVID-19 (5). The fear of COVID-19 and its serious consequences (miscarriage, premature birth, low birth weight, stillbirth, and neonatal death) (5, 6) can cause anxiety and depression among pregnant women. This situation may worsen among pregnant women in the early pregnancy (i.e., <13 gestational weeks), a period critical for fetal development, and pregnant women re-adjusting their physical and psychological changes. During the pandemic period, even if pregnant women are not infected with COVID-19, various prevention and pre-pandemic measures could still affect daily life, antenatal care, and accompanying childbirth arrangements, increasing their psychological burden. The COVID-19 vaccination program does not involve pregnant women in China, worsening their psychological status. Anxiety and depression in pregnancy affect the pregnancy outcome (premature birth and low birth weight) and the social and emotional development, cognitive and language development, exercise ability, and adaptive behavior of offspring (7). Further, strict public health measures in response to the pandemic, such as quarantine, regular PCR test, and lockdown, may increase the mental health burden of pregnant women as they have less accessibility to routine care as needed (8). Therefore, under the current COVID-19 pandemic, it is essential to understand the psychological issues of pregnant women during the pandemic period to provide tailored services to mitigate the impact.

Anxiety and depression are two of the most common mental health conditions (9). Anxiety involves excessive worrying or stress about the outcome of a situation. Depression, on the other hand, is a mood disorder that causes overwhelming feelings of sadness or apathy (10). Previous studies conducted in China demonstrated an increased risk of anxiety and depression among pregnant women, especially in the early phase of the pandemic (11–14). However, whether the burden of anxiety and depression is reduced after the easing of COVID-19 measures is still not clear, while understanding this is essential in planning mental health support services for pregnant women.

This pre-post study aimed to fill the research gap by evaluating the impact of COVID-19 on the mental health of Chinese pregnant women by comparing the burden of depression and anxiety symptom severity before and after the pandemic outbreak. Primarily, we wanted to measure whether the psychological status would back to normal after the ease of the COVID-19 measures.

## Methods

This study is the baseline survey of a birth cohort study. This study was registered to the Chinese Clinical Trial Registry (ChiCTR2000029022), and ethical approval was obtained from Shanghai Ethics Committee for Clinical Research (SECCR/2019-43-01). All methods were performed following the relevant guidelines and regulations. Informed consent was obtained from all participants. Pregnant women 18-year-old or older who visited Fenglin Community Health Service Center (CHC) in Shanghai to make a pregnancy register between September 29, 2019, and November 5, 2020, were eligible and recruited. The participants were assessed immediately after the recruitment. By November 5, 51,177,514 confirmed COVID-19 cases were identified and reported globally, while 1,345,741 deaths were recorded.

### Measures

Participants were asked to complete the study survey electronically (by scanning a QR code) to collect information on age, gestational week, the number of fetuses, conception mode, education level, occupation, height, weight, etc. Patient Health Questionnaire-9 items (PHQ-9), a nine-item questionnaire designed to screen for depression, was used to measure whether a participant had depression. PHQ-9 measures depression based on the frequency of symptoms in nearly 2 weeks and is graded at 0–3 for each item, with a total score range of 0–27. It can be used to assess the severity of depressive symptoms: 0–4 depression-free, 5–9 mild, 10–14 moderate, and 15 or above severe. PHQ-9 has good confidence and is the most common tool for screening depression in primary health care institutions at home and abroad (15, 16). Generalized Anxiety Disorder Scale-7 items (GAD-7), a seven-item questionnaire designed to screen for anxiety, was also used in our survey. GAD-7 has seven items, and each can be graded from 0 to 3, with a total score range of 0–21. It can be used to assess the severity of anxiety symptoms: 0–4 anxiety-free, 5–9 mild, 10–14 moderate, 15 or above severe. Studies support the reliability and validity of the GAD-7 (15, 17). In this study, five was used as a cutoff score to determine both anxiety and depression (15). Internal reliability was excellent for both scales (Cronbach's alpha = 0.89 for PHQ-9; Cronbach's alpha = 0.92 for GAD-7) (15). In our study, we presented mild, moderate, and severe depression and anxiety, as we not only wanted to identify pregnant women who should be clinically diagnosed with depression and anxiety but also wanted to identify people who are at high risk of these two mental health disorders to provide timely supporting services, especially at the early gestational weeks of the pregnancy.

### Participants categorization

This study is the baseline survey of a birth cohort, while only the baseline data were used in the data analysis. However, based on the time the participants were recruited, we categorized the participants into pre-pandemic and pandemic groups. Participants recruited before January 23, 2020 (The date that China issued the first-grade public health response toward COVID-19) were categorized into the pre-pandemic group. The participants recruited after this time point were classified into the pandemic group. Overall, around one-quarter of the participants were categorized into the pre-pandemic group, and three-quarters were categorized into the pandemic group. We further categorized the pandemic group into the outbreak and back-to-normal groups, with the breaking point of May 11, 2020, when Shanghai started to ease the COVID-19 measures. There were also no community-transmitted COVID-19 cases in Shanghai during the back-to-normal study period. Reporting the mental health status of pregnant women during the period of back-to-normal would be essential in planning future intervention programs.

### Data analysis

The participants filled out the survey electrically, and the gynecologist immediately reviewed the questionnaire. Continuous variables were presented as means and standard deviations or median and interquartile ranges. Descriptive statistics were used to describe the sociodemographic information of the participants. The Mann-Whitney *U* and chi-square tests were used to compare the differences between the study groups. Multivariable logistic regression was used to determine the factors associated with depression and anxiety in pregnant women. We used enter method for variable selection; only variables with *P* <0.10 were included in the final model. *P* < 0.05 was considered as the differences statistically significant. Analyses were conducted using SPSS 26.0.

## Results

### Sociodemographic characteristics

A total of 478 pregnant women were included in this study. There were 113 in the pre-pandemic group and 365 in the pandemic group. The participants were 31 ± 4 years old, and their Body Mass Index (BMI) was 21.4 ± 3.0 kg/m^2^. The demographic characteristics of the two study groups are shown in Table 1. There was no statistical difference in age, BMI, nationality, occupation, parity, the number of fetuses, and the conception mode of the two groups. However, the two groups differed in educational level and gestational week.

**Table 1:** Descriptive analysis of the demographic characteristics, 2019–2020 (*N* = 478).

**Characteristic**	**Pre-pandemic group (*n* = 113)**	**Pandemic group (*n* = 365)**	**Chi-square**	***P***	**Outbreak group (*n* = 125)**	**Back-to-normal group (*n* = 240)**	**Chi-square**	***P***
	**September 29, 2019–January 23, 2020**	**January 31, 2020–November 5, 2020**			**January 31, 2020–May 11, 2020**	**May 12, 2020–November 5, 2020**		
	***n* (%)**	***n* (%)**			***n* (%)**	***n* (%)**		
**Age**			2.111	0.15			0.136	0.71
<35 years old	97 (85.8)	291 (79.7)			101 (80.8)	190 (79.2)		
≥35 years old	16 (14.2)	74 (20.3)			24 (19.2)	50 (20.8)		
**BMI before pregnancy**			0.952	0.62			1.363	0.51
<18.5	13 (11.5)	55 (15.1)			19 (15.2)	36 (15.0)		
18.5–23.9	82 (72.6)	251 (68.8)			82 (65.6)	169 (70.4)		
≥24	18 (15.9)	59 (16.1)			24 (19.2)	35 (14.6)		
**Nation**			1.662	0.20			0.000	1.00
Han	113 (100.0)	356 (97.5)			122 (97.6)	234 (97.5)		
Ethnic minority	0 (0.0)	9 (2.5)			3 (2.4)	6 (2.5)		
**Educational level**			6.754	0.034			5.576	0.062
High school or below	12 (10.6)	34 (9.3)			14 (11.2)	20 (8.3)		
Bachelor degree	81 (71.7)	221 (60.5)			83 (66.4)	138 (57.5)		
Master degree or above	20 (17.7)	110 (30.1)			28 (22.4)	82 (34.2)		
**Occupation**			4.634	0.33			6.158	0.19
Professional	36 (31.9)	113 (31.0)			30 (24.0)	83 (34.6)		
Clerk	26 (23.0)	101 (27.7)			35 (28.0)	66 (27.5)		
Commercial service personnel	30 (26.5)	78 (21.4)			31 (24.8)	47 (19.6)		
Other occupation	19 (16.8)	53 (14.5)			23 (18.4)	30 (12.5)		
Unemployed	2 (1.8)	20 (5.5)			6 (4.8)	14 (5.8)		
**Parity**			0.029	0.87			1.608	0.21
Primiparity	88 (77.9)	287 (78.6)			103 (82.4)	184 (76.7)		
Multiparity	25 (22.1)	78 (21.4)			22 (17.6)	56 (23.3)		
**Number of fetuses**			0.624	0.43			1.967	0.16
Single birth	112 (99.1)	355 (97.3)			119 (95.2)	236 (98.3)		
Twins	1 (0.9)	10 (2.7)			6 (4.8)	4 (1.7)		
**Mode of conception**			2.754	0.097			0.265	0.61
Naturally reproduction	108 (95.6)	331 (90.7)			112 (89.6)	219 (91.3)		
Assisted reproduction	5 (4.4)	34 (9.3)			13 (10.4)	21 (8.8)		
**Gestational week**			4.959	0.026			0.183	0.67
<13 week	99 (87.6)	285 (78.1)			96 (76.8)	189 (78.8)		
≥13 week	14 (12.4)	80 (21.9)			29 (23.2)	51 (21.3)		

### Psychological scales

The median depression scale score in the pre-pandemic group was 3.0 (6.0), and the median anxiety scale score was 0.0 (2.0). The median depression scale score was 4.0 (5.0) for the pandemic group, and the median anxiety scale score was 0.0 (3.0). The mean depression scale score in the pre-pandemic group was 4.34 ± 4.78, and the mean anxiety scale score was 1.56 ± 2.60. The mean depression scale score was 5.04 ± 4.20 for the pandemic group, and the mean anxiety scale score was 1.90 ± 3.06.

With a depression cutoff score of 5 for PHQ-9, 40 (35.4%) pregnant women were considered depressed in the pre-pandemic group, and 174 (47.7%) participants were supposed to be depressed in the pandemic group. The depression rate was higher in the pandemic than in the pre-pandemic group. With an anxiety cutoff score of 5 for GAD-7, 15 (13.3%) pregnant women were anxious in the pre-pandemic group, and 59 (16.2%) people were anxious in the pandemic group. The rates were similar in the two study groups. Besides, 12.9% of the participants in the pandemic group were in moderate and severe depression (PHQ-9 ≥ 10). However, only 3.0% of the participants in the pandemic period had moderate and severe anxiety (GAD-7 ≥ 10). Detailed scores are presented in Table 2.

**Table 2:** Distribution of scores on the psychological scales, 2019–2020 (*N* = 478).

**Scales**	**Pre-pandemic group (*n* = 113)**	**Pandemic group (*n* = 365)**	**Outbreak group (*n* = 125)**	**Back-to-normal group (*n* = 240)**
	***n* (%)**	***n* (%)**	***n* (%)**	***n* (%)**
**PHQ-9**
0–4 (depression-free)	73 (64.6)	191 (52.3)	74 (59.2)	117 (48.8)
5–9 (mild depression)	27 (23.9)	127 (34.8)	40 (32.0)	87 (36.3)
10–14 (moderate depression)	9 (8.0)	36 (9.9)	8 (6.4)	28 (11.7)
15–27 (severe depression)	4 (3.5)	11 (3.0)	3 (2.4)	8 (3.3)
**GAD-7**
0–4 (anxiety-free)	98 (86.7)	306 (83.8)	110 (88.0)	196 (81.7)
5–9 (mild anxiety)	14 (12.4)	48 (13.2)	10 (8.0)	38 (15.8)
10–14 (moderate anxiety)	0 (0.0)	7 (1.9)	3 (2.4)	4 (1.7)
15–21 (severe anxiety)	1 (0.9)	4 (1.1)	2 (1.6)	2 (0.8)

After further categorizing the pandemic group into the outbreak and back-to-normal group, there were no statistical differences in age, BMI, nationality, occupation, education, gestational week, parity, fetal number, and conception mode between the two groups (Table 1). Overall, 51 (40.8%) pregnant women were in depression in the outbreak group, and 123 (51.3%) participants were in depression in the back-to-normal group. The Mann-Whitney *U*-test showed the median depression scale score of the back-to-normal group [5.0 (5.75)] was statistically higher than that in the outbreak group [4.0 (4.5)] (*P* = 0.028). 18.3% of people were considered anxious in the back-to-normal group, and 12.0% were supposed to be anxious in the outbreak group. The percentages were similar in the two study groups.

### Factors associated with depression and anxiety

The results of the multivariable analysis indicated that participants with a gestational week of fewer than 13 weeks were more likely to have depression, with an adjusted OR (aOR) of 1.81 (95% CI:1.11–2.95). In addition, participants recruited during the pandemic period were more likely to be determined to be in depression, with aOR of 1.81 (95% CI: 1.16–2.84). There were no statistical differences in anxiety status before and after the outbreak of COVID-19 among pregnant women (aOR = 1.27, 95% CI 0.68–2.36). Details are presented in Table 3.

**Table 3:** Logistic regression analysis of depression and anxiety, 2019–2020 (*N* = 478).

	**Depression**		**Anxiety**	
	**Adjusted OR (95%CI)**	***P***	**Adjusted OR (95%CI)**	***P***
**Mode of conception**
Naturally reproduction	Reference		Reference	
Assisted reproduction	0.78 (0.40–1.54)	0.48	0.58 (0.20–1.70)	0.32
**Educational level**
High school or below	Reference		Reference	
Bachelor degree	1.44 (0.74–2.82)	0.28	1.27 (0.50–3.22)	0.62
Master degree or above	1.26 (0.62–2.59)	0.52	1.37 (0.51–3.69)	0.53
**Gestational week**
<13 week	1.81 (1.11–2.95)	0.017	0.98 (0.52–1.84)	0.94
≥13 week	Reference		Reference	
**COVID-19 effect**
Pre-pandemic	Reference		Reference	
Pandemic	1.81 (1.16–2.84)	0.009	1.27 (0.68–2.36)	0.45

## Discussion

Our study indicated that the risk of depression significantly increased among pregnant women after the pandemic. Our finding is consistent with a multi-centered study conducted in 10 Chinese provinces, which reported an increased depression risk in pregnant women after the outbreak of COVID-19 (18). Several reasons could explain this situation. On the one hand, pregnant women may worry about COVID-19's effects on their health and offspring, increasing their risk of depression. Pregnant women would fear that the COVID-19 infection will likely affect placental function (6, 19) and seriously impact offspring, mainly abortion, premature birth, fetal embarrassment, low birth weight, fetal death, and neonatal death (5, 6). On the other hand, the COVID-19 measures seriously impact pregnant women's daily life and routine antenatal examination, exposing them to the stress of “the pandemic is around the corner,” which further increases their risk of depression.

We found that the psychological status of pregnant women did not improve during the back-to-normal period. Several reasons would have led to this phenomenon. First, the pandemic has not completely subsided globally, leading to the concern that the pandemic may come back to Shanghai anytime. Even though Shanghai and the whole of China had recovered from the pandemic since then, the international pandemic was intensifying. As a port city, Shanghai continues to have imported cases, making pregnant women think that the pandemic is still there. Second, the prevention and control measures had not been erased during the back-to-normal period, which continues to impact the daily life of pregnant women. For example, reducing the amount of prenatal exercise can increase the risk of prenatal depression (20). Thirdly, the future direction of the pandemic is uncertain, and the ending time of control measures is not determined, so neonatal protection will make mothers feel overwhelmed after delivery. Therefore, under the normalization of pandemic prevention and control, we should actively pay more attention to pregnant women's psychological status to reduce the long-term impact on maternal psychology and help pregnant women recover from the pandemic.

The study also found that pregnant women in early pregnancy have an increased risk of depression. This finding is also consistent with a survey conducted in Shanxi, China (21). This study reported that depression in early pregnancy was about 47.3%, much higher than 34.0% in middle and late pregnancy. Early pregnancy is a critical period for fetal development. Pregnant women in early pregnancy experience intensive diet, behavioral activities, and health care changes, which cause psychological imbalance. Some pregnancies are accidental, and the feelings of powerlessness in living change lead to a growth in depression.

Our study indicated that after the outbreak of COVID-19, 12.9% of the participants were in moderate and severe depression (PHQ-9 ≥ 10). Even though this prevalence is lower than the findings of a study conducted in China before the pandemic (16.3%), it is still very high (22). The high, moderate, and severe depression rate is a risk signal, and timely and effective health education could help pregnant women cope with their challenges. Thus, prenatal clinics should offer tailored services to help pregnant women handle adverse situations and improve their psychological status during pregnancy. At the same time, it is necessary to carry out psychological state evaluation among pregnant women and make intervention timely. CHCs can provide psychological therapists to pay more attention to mental health during the maternal period and carry out tertiary prevention according to “Consensus on maternal mental health management (2019)” (23), compiled by the Psychosomatic Health Society of China Preventive Medicine Association, and Committee of Women Mental Health Care of China Maternal and Child Health Association.

Our study further indicated that the anxiety level did not increase much since the pandemic, and it stayed the same after the ease of COVID-19 measures. This finding is different from the results of depression, which may reflect the fact that depression and anxiety capture the different points of mental health disorders among pregnant women. In addition, only 3% of the participants were in moderate and severe anxiety, much lower than the findings of a systematic review and meta-analysis, which reported a pooled prevalence for a clinical diagnosis among pregnant women of any anxiety disorder of 15.2% (24).

Our study has several limitations. First, this study's sample size is limited, and the participants are pregnant women from Fenglin Community, which cannot represent Shanghai as a central part. Second, the factors influencing the mental health status included in this study are not yet comprehensive, and many potential confounders were not considered. Third, although the psychological assessment scales (PHQ-9 and GAD-7) used in this study are internationally recommended, the self-reported scales cannot be diagnostic because of subjective effects. Last but not least, this study is cross-sectional, and our study may suffer from selection bias. It can only determine the association between depression, anxiety, and potential risk factors (25).

In conclusion, pregnant women in Shanghai have an increased risk of depression after the COVID-19 outbreak, which is not decreased after the ease of COVID-19 measures. Maternal health care institutions should pay close attention to the long-term impact on pregnant women's psychology under the COVID-19 pandemic, strengthen the promotion of the mental health of pregnant women, monitor the psychological status of pregnant women, offer timely intervention, and carry out an effective referral.

## Data availability statement

The raw data supporting the conclusions of this article will be made available by the authors, without undue reservation.

## Ethics statement

Ethical approval was obtained from Shanghai Ethics Committee for Clinical Research (SECCR/2019-43-01). The patients/participants provided their written informed consent to participate in this study.

## Author contributions

JZ and WT conceived the idea and analyzed the data. JZ, HY, LX, and CY collected the data. JZ and WT drafted the manuscript, while HY, LX, and CY commented on the manuscript. All authors contributed to the article and approved the submitted version.

## Funding

The authors thank the Youth Project of Shanghai Xuhui District Health Commission and Xuhui District Science and Technology Commission (SHXH201948), and the Joint Research Project of Shanghai Xuhui District Health Commission (XHLHGG201805) for their support.

## Conflict of interest

The authors declare that the research was conducted in the absence of any commercial or financial relationships that could be construed as a potential conflict of interest.

## Publisher's note

All claims expressed in this article are solely those of the authors and do not necessarily represent those of their affiliated organizations, or those of the publisher, the editors and the reviewers. Any product that may be evaluated in this article, or claim that may be made by its manufacturer, is not guaranteed or endorsed by the publisher.
