# Corrigendum: The impact of COVID-19 on the mental health of pregnant women in Shanghai, China

**DOI:** 10.3389/fpubh.2024.1491504

**Published:** 2024-10-18

**Authors:** Jiali Zhang, Hualong Yuan, Liping Xu, Chuntao Yi, Weiming Tang

**Affiliations:** ^1^Fenglin Community Health Service Center, Shanghai, China; ^2^Guangdong Second Provincial General Hospital, Guangzhou, China; ^3^The University of North Carolina at Chapel Hill Project-China, Guangzhou, China

**Keywords:** depression, anxiety, pregnant women, COVID-19, China

In the published article, there was an error in affiliation 2. Instead of “Guangdong No. 2 Provincial People's Hospital, Guangzhou, China”, it should be “Guangdong Second Provincial General Hospital, Guangzhou, China”.

The authors apologize for this error and state that this does not change the scientific conclusions of the article in any way. The original article has been updated.

